# Estimation of cAMP binding in hippocampus CA1 field by a fluorescent probe

**DOI:** 10.3389/fcell.2023.1267956

**Published:** 2023-09-29

**Authors:** Carla Mucignat-Caretta, Antonio Caretta

**Affiliations:** ^1^ Department of Molecular Medicine, University of Padova, Padova, Italy; ^2^ Department of Food and Drug Science, University of Parma, Parma, Italy

**Keywords:** cAMP, PKA, hippocampus, development, fluorescent probe

## Abstract

The hippocampus is an allocortex structure involved in many complex processes, from memory formation to spatial navigation. It starts developing during prenatal life but acquires its adult functional properties around the peripubertal age, in both humans and mice. Such prolonged maturation is accompanied by structural changes in microcircuitry and functional changes involving biochemical and electrophysiological events. Moreover, hippocampus undergoes plasticity phenomena throughout life. In murine rodents, the most relevant maturation steps in Cornu Ammonis 1 (CA1) hippocampal subfield occur during the third-fourth weeks of life. During this period, also the expression and localization of cAMP-dependent protein kinases (PKA) refines: many regulatory (R1A) PKA clusters appear, bound to the cytoskeleton. Here the binding characteristics of R1A are determined in CA1 by using confocal microscopy. Apparently, two binding sites are present with no evidence of cooperativity. Equilibrium dissociation constant is estimated around 22.9 nM. This value is lower from that estimated for R1A in soluble form, suggesting a different binding site conformation or accessibility in the tissue. The method described here may be useful to track the developmental changes in binding activity, which affects cAMP availability at selected intracellular microzones. Possible relations with functional consequences are discussed.

## 1 Introduction

Mammalian hippocampus is involved in a variety of functions crucial for survival, like spatial navigation and memory, but also in the processing of social cues and emotional reactivity, including fear. Its shape is similar among mammals, including a C-shaped Cornu Ammonis (CA) and a V-shaped dentate gyrus (DG), yet homology is apparent also with sauropsid brain, as confirmed by gene expression and functional studies (Medina et al., 2017).

From the seminal work of Lorente de Nò (1934), the hippocampal formation has been subdivided in 4 CA different subfields, numbered from CA1 to CA4, in addition to the DG (Borzello et al., 2023). Hippocampal pyramidal and granule neurons share a lineage with cortical and diencephalic neurons, while hippocampal GABAergic interneurons have distinct progenitors (Martin et al., 2002). Besides structural and anatomical differences with the other CA fields, including smaller pyramidal neurons with dendritic spines and specific input/output connections, CA1 has exclusive biochemical, molecular and electrophysiological properties, including sensitivity to long-term potentiation (LTP), to epilepsy and to ischemic insults (Lein et al., 2004; Dudek et al., 2016; Lin et al., 2018). The local circuits within CA1 are particularly important for memory and spatial navigation (Geiller et al., 2023), while downregulation of GABAergic inhibition in CA1 results in epileptiform electrical activity (Liu et al., 2014).

Pyramidal cells in CA1 show different electrical activity and reactivity throughout development (McKiernan and Marrone, 2017). Gamma oscillatory activity driven by basket cells changes in shape in the period between postnatal day (PN) 6 and PN25, when their activity turns from slow to fast (Doischer et al., 2008). At variance with CA2, synapses in CA1 are vigorously modified by LTP (Carstens and Dudek, 2019), that strengthens glutamatergic signaling for contextual learning, with modifications in the balance between excitation and inhibition (Sakimoto et al., 2021).

Different cell subtypes may drive the various activities, for example, basket cells will reach the adult number in the third PN week but only during the fourth they will mature (Donato et al., 2015), peaking parvalbumin expression in CA1 by PN26, after the synaptogenesis peak at P23 (Donato et al., 2017). Also, from PN17 to PN 24 there is a decrease in phospho-CREB, while other kinases increase, in parallel with establishment of hippocampal circuits (Travaglia et al., 2016).

Electrical activity in spatial navigation neurons appears during the second-third PN week, while during the fourth week periodic firing patterns emerge (Langston et al., 2010). Functionally, CA1 properties for memory consolidation appear from the beginning of the fourth PN week (Farooq and Dragoi, 2019) with a limited environmental sensitivity (Figurov et al., 1996; Gao et al., 2018). A similar prolonged post-natal development is seen also in humans (Hachiya and Takashima, 2001). Rapid transcriptomic changes emerge between PN21 and PN25 (Que et al., 2021) which parallel functional changes. They are similar to imprecise contextual fear memories in PN16-PN20 mice that mature along with perineuronal networks around PN24 in CA1, and around PN16 in CA3 and dorsal DG (Ramsaran et al., 2023). All these data point to relevant changes in CA1 between the third/fourth PN week. The cyclic nucleotide signaling pathway is involved in hippocampal synaptic transmission, neuron excitability, plasticity and neuroprotection (Heckman et al., 2018).

Our laboratory clarified that during development, both CA hippocampal subfields and dentate gyrus undergo remarkable changes in the expression and localization of the four cAMP-dependent protein kinases (PKA) regulatory subunits, named PKA R1Alpha (R1A), R1Beta (R1B), R2Alpha (R2A) and R2 Beta (R2B). Briefly, R2B is the most abundant PKA subunit in the brain before birth and at birth is present in the whole hippocampus, mainly on the CA pyramidal layer and on dentate gyrus granular layer, apparently increasing in the first postnatal week. In the subsequent week, R2B declines only in CA1, where it is substituted by R1A, while R2B still persists on CA2-CA3-CA4 (Mucignat-Caretta and Caretta, 2004). Taking advantage of a fluorescently-tagged cAMP analogue (Mucignat-Caretta and Caretta, 1997) in addition to immunofluorescence, we showed that highly concentrated R1A aggregates appear around PN8 in the granular layer of dentate gyrus and around PN10 in CA1 as small punctuated pattern, they increase in CA1 by PN 20-25 while in dentate gyrus they decrease from PN30 onwards, and only remain in CA1 of 2 years old rats (Mucignat-Caretta and Caretta, 2002). This finding was quite puzzling since in solution fluorescent cAMP did bind well to both R1 and R2 (Mucignat-Caretta and Caretta, 1997), while in the brain it binds only to R1A aggregates, suggesting a different conformation or accessibility of the cAMP binding site *in vivo*, at specific developmental times and locations. Throughout development and aging, by both immunohistochemistry, immunofluorescence and equilibrium binding (Mucignat-Caretta et al., 1999), R1A aggregates were not found in CA2, and only transiently around the third-fourth postnatal weeks in CA3-CA4. Differences in expression/localization of cAMP-binding PKA may result in different buffering activity and in variations in local cAMP concentration at specific subcellular sites, with functional consequences on a variety of downstream processes. Contrary to R1A widespread distribution all over the brain when in soluble form, R1A aggregates are found in the neuronal detergent-insoluble fraction at particular ages and at specific sites only, where they are bound to cytoskeleton (Mucignat-Caretta et al., 1999). In CA1 it partially co-localizes with PKA catalytic subunit (Mucignat-Caretta and Caretta, 2020). Notably, three laboratories using two different technologies reported higher R1A and R1B expression, when compared to R2A and R2B in the whole hippocampus (see Mucignat-Caretta and Caretta, 2020). However, no data are available on the possible differences in cAMP binding at different sites in the brain tissue *in situ*, which may result in different activation kinetics of PKA.

The aim of this work is to provide a reliable method for the determination of cAMP binding by R1A in CA1 using confocal microscopy, which could be exploited to determine developmental changes and trace its functional consequences.

## 2 Materials and methods

### 2.1 Animals

Ten Fischer rats and ten CD-1 mice, 30 to 35 PN days old were reared in the inhouse animal facility on 12:12 h light cycle, with rodent chows and tap water *ad libitum*, at 23°C ± 1°C. Animals were anesthetized with halothane before cervical dislocation. Brains were immediately removed and frozen in nitrogen-cooled pentane, cut on a cryostat (Bright Clinicut) in coronal or horizontal 16 μm sections. Chemicals were from Sigma (Milan, Italy), unless otherwise stated.

### 2.2 Immunofluorescence

Sections were washed in 100 mL phosphate-buffered saline (PBS: 150 mM NaCl, 15 mM phosphate buffer, 0.5 mM ethylene-diaminetetraacetic acid, pH 7.4) for 30 min at 25°C, fixed in formalin (10% in PBS) for 1 h, rinsed in PBS-TritonX-100 2% for 30 min.

After blocking non-specific binding (30 min with 0.4% bovine serum albumin in PBS), alternate sections were incubated with rabbit anti-RI, RII, Catalytic subunit or tyrosine hydroxylase (respectively: sc-907, sc-909, sc-903, sc-14007 Santa Cruz Biotechnology, Santa Cruz, CA) antibodies 1:200 in PBS, at room temperature overnight. After washing in PBS, sections were incubated at 37°C for 30 min with either Alexa 488- or Alexa 594-conjugated anti-rabbit secondary antibody (Molecular Probes, Eugene, OR), 1:250 in PBS.

Colocalization with DAPI and FITC-labelled bungarotoxin: sections were fixed in formalin for 1 min at 37°C and incubated with 200 nM Alexa 488-cAMP (Molecular Probes, Eugene, OR), or 100 nM 8-(5-thioacetamidofluorescein)-adenosine 3′,5′-cyclic monophosphate (SAF-cAMP) or 8-(5-thioacetamidotetramethylrhodamine)-adenosine 3′,5′-cyclic monophosphate (SAR-cAMP, Mucignat-Caretta and Caretta, 2001). These three fluorescent cAMP molecules gave reproducibly overlapping results. Controls were run according to Mucignat-Caretta and Caretta, 2020, additional controls are presented in Caretta et al., 1991; Ribaudo et al., 2020. Selected sections were Nissl stained.

Slides were examined with an epifluorescence microscope (Leica DMR), objectives 20×, 40×, and 100× (oil immersion, numerical aperture 1.30). Photographs (768 × 582 voxels) were acquired with the resident software and handled with Graphic Converter 9 for superimposition.

### 2.3 Saturation and competitive binding experiments

Binding experiments were done on P30 rat brains. Coronal sections (−2.80 to −3.80 mm from Bregma) were fixed for 30 s in formalin with TritonX-100 2% in PBS at 37°C, and rinsed. For saturation binding experiments, they were covered with SAF-cAMP in PBS at different dilutions, as indicated. CA1 was visualized with Sarastro confocal microscope using a ×20 objective, laser λex 488 nm at 100% power for 10 μsec, emission beamsplitter and filter 510 nm. A vertical scan (128 voxels × 0.6 μm) was used to set the depth of the scan in the middle of the tissue section. One single horizontal scan (1,024 voxels × 0.6 μm) in each section was taken to avoid fluorescein photobleaching. Two sections were examined at each concentration at a power giving non-saturating values (below 255, typically in the range around 80–150 arbitrary units) to preserve linearity of photomultiplier tube (PMT) readouts. The most intense voxel value in the 40 most intense dots were measured with the resident software, of which only the 15 most intense were averaged. Since the most intense structures could span over more than one single voxel, it was not possible to rely only on the most intense voxel histograms, hence it was necessary to measure only the most intense voxel at each labelled structure. The intensity value included SAF-cAMP fluorescence but also the fluorescence of the tissue itself, hence the average value of the tissue background around the labelled hippocampus (3 unlabelled rectangles, above and below the labelled area in the pyramidal layer), devoid of any labelled structure, was subtracted from the calculated mean intensity. The resulting value was related to the calibration curve, obtained using fluorescein solutions (3,000 and 6,000 nM), scanned at each of the 16 PMT gain/laser power combinations that were chosen to give non-saturating voxel values, by measuring the average intensity at three different areas in each of two different microscope slides loaded with 30 μL fluorescent solution (24 × 32 mm coverslips). The 6 values were averaged and used to calculate the concentration of SAF-cAMP in the maxima.

Inhibition curves were generated in competitive binding experiments by adding increasing quantities of unlabeled cAMP to displace SAF-cAMP at a fixed concentration. Slides were incubated for 5 min in PBS, IBMX 500 μM SAF-cAMP 88 nM with the required cAMP concentration (from 0 to 3,000 nM) and examined as above.

### 2.4 Data analysis

Experiments were replicated at least three times, the figures show representative images. ImageJ was used to extract from conventional (non-confocal) fluorescence images the number of pixels at each intensity, and the number and location of maxima (defined as pixels 30 fluorescence units above the adjacent, according to Mucignat-Caretta and Caretta, 2020). Binding curves were generated and analyzed with GraphPad Prism 9.

## 3 Results

### 3.1 Description of insoluble regulatory and catalytic PKA distribution in CA1 and their binding to fluorescent cAMP

**FIGURE 1:**
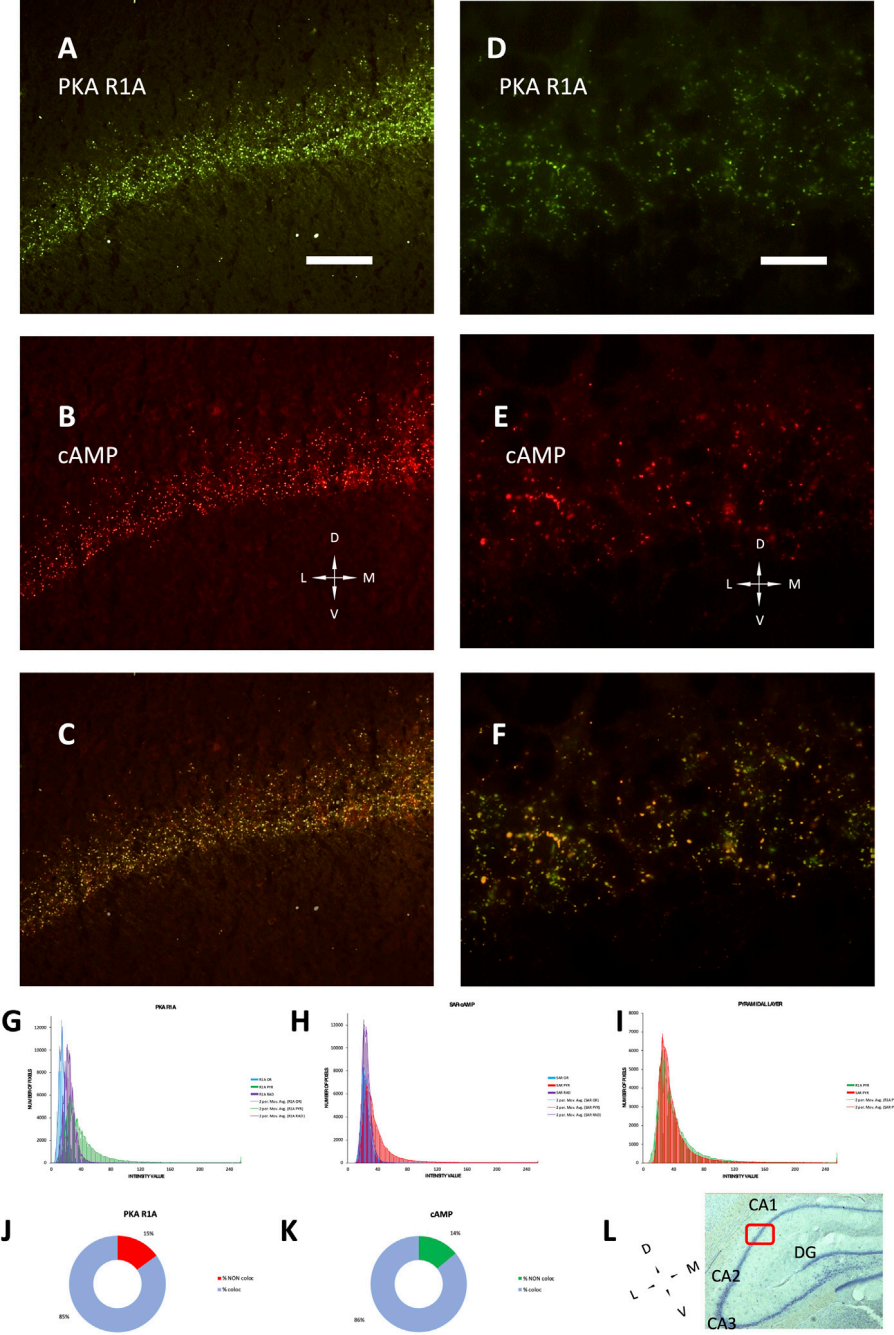
PKA R1A immunolabelling and SAR-cAMP equilibrium binding, mainly localized in the pyramidal layer, superimpose in P30 rat CA1 by conventional epifluorescence microscopy. **(A–C)**: low-power (20× objective) micrograph of the same field. **(A)** R1A immmunolabeling. **(B)** SAR-cAMP. **(C)** merge of A and B, showing colocalization of the two signals. **(D–F)** high-power (100× objective) micrograph of the same field. **(D)** R1A immmunolabeling. **(E)** SAR-cAMP. **(F)** merge of D and E, showing colocalization of the two signals. Coronal sections; D, dorsal; L, lateral; M, medial; V, ventral; Scale bar = 50 **(A–C)** and 10 μm **(D–F)**. **(G–I)**: distribution of intensity in the 3 hippocampal layers: stratum oriens (OR, blue), pyramidale (PYR, green in **(G,I)**, red in **(H,I)** and radiatum (RAD, violet). Bars are connected by moving mean solid lines of the same color. **(G)** distribution of PKA R1A intensity labelling in A. **(H)** distribution of SAR-cAMP intensity labelling in B. **(I)** superimposition of PKA R1A (green bars) and SAR-cAMP (red bars) intensity labelling in the stratum pyramidale. **(J,K)**: percentage of maxima co-localizing or not in **(F)**. **(J)**: percentage of PKAR1A maxima colocalizing (light blue) or not (red) with SAR-cAMP. **(K)**: percentage of SAR-cAMP maxima colocalizing (light blue) or not (green) with PKAR1A. **(L)**: Nissl-stained section of a rat brain (5× objective), showing the localization of area 1B of CA1 (red box), in which the experiments and photographs were made. Dorsal on the top. CA1-2-3: Cornu Ammonis hippocampal subfields 1-2-3. DG, Dentate gyrus.

**FIGURE 2:**
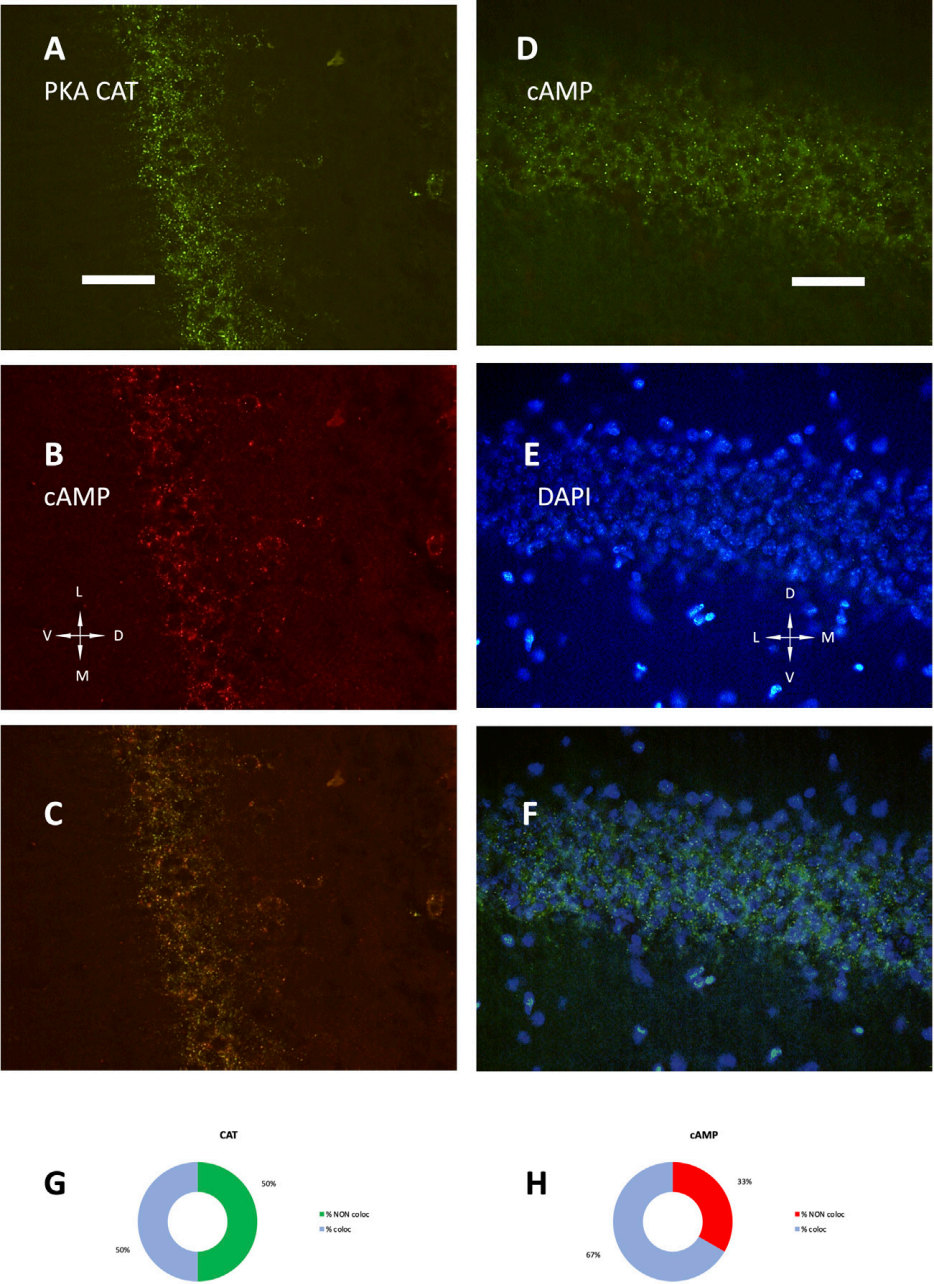
**(A)** PKA catalytic subunit immunolabeling. **(B)** SAR-cAMP, same field as **(A)**. **(C)** merge of **(A,B)**. **(D)** SAF-cAMP equilibrium binding. **(E)** DAPI, same field as **(D)**. **(F)** merge of **(D,E)**. Coronal sections, P35 mouse, 40× **(A–C)** and 100× **(D–F)**. D, dorsal; L, lateral; M, medial; V, ventral. Scale bar = 25 **(A–C)** and 10 μm **(D–F)**. **(G**,**H)** quantification of signal colocalization in **(C)**. **(G)** percentage of PKA catalytic subunit maxima colocalizing (light blue) or not (green) with SAR-cAMP. **(H)** percentage of SAR-cAMP maxima colocalizing (light blue) or not (red) with PKA catalytic subunit.

**FIGURE 3:**
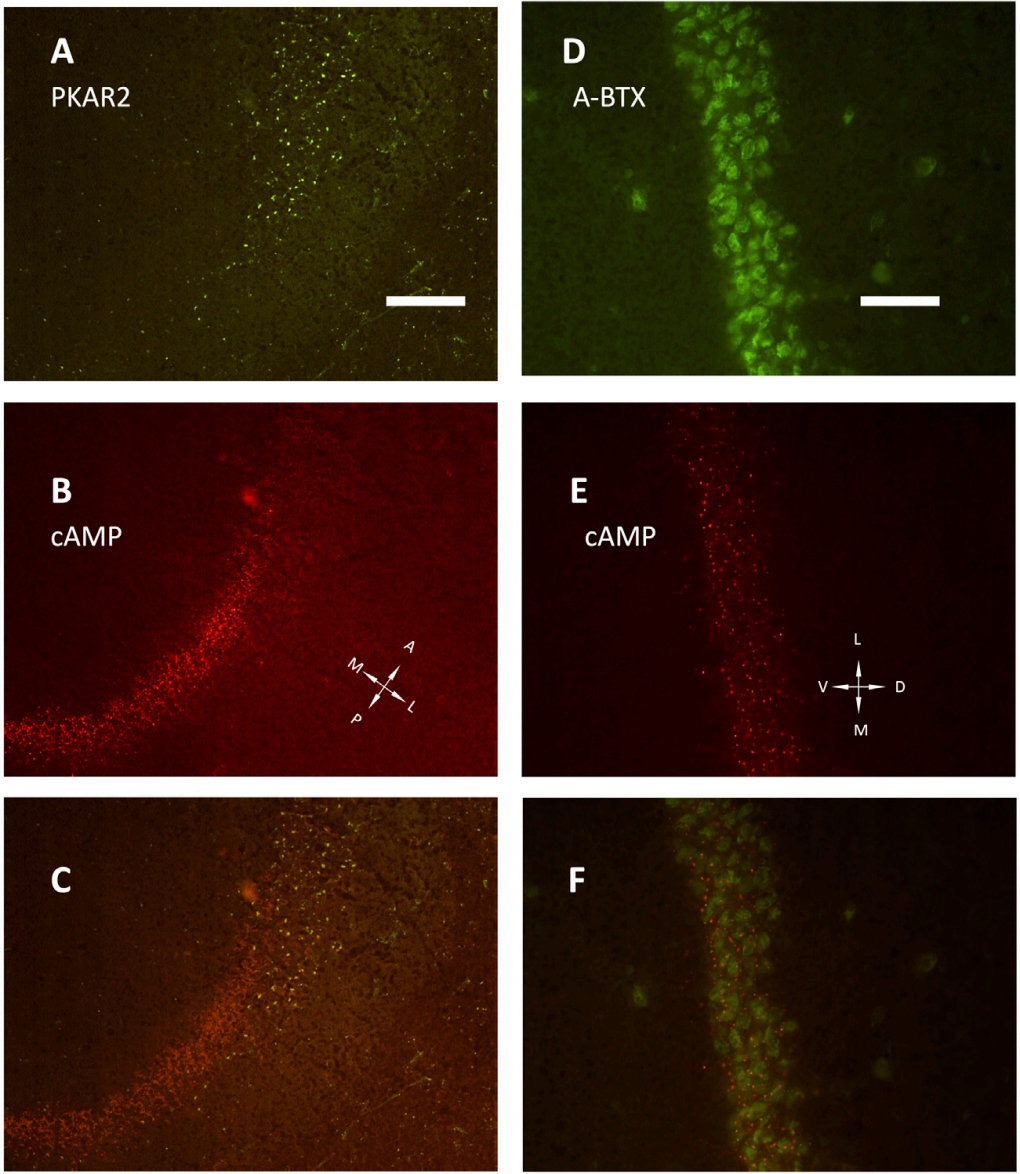
**(A–C)** low power image (20× objective) of the transition between CA1 and CA2 fields, horizontal section. **(A)** PKA R2 immunolabelling (green). **(B)** SAR-cAMP, same field as **(A)**. **(C)** merge of **(A,B)** the two signals appear localized in different areas, being PKA R2 present as scattered in stratum oriens (right side of the image) and radiatum (left side of the image), while the pyramidal layer is labelled only in CA2 (upper part of the image). At variance, SAR-cAMP labelling (B, red) appears limited to the CA1 pyramidal layer. **(D–F)**: CA1 pyramidal layer, coronal section (40× objective). **(D)** alpha-bungarotoxin (green, A-BTX) labeling. **(E)** same field as D, SAR-cAMP. **(F)** merge of **(D,E)**. A, anterior; D, dorsal; L, lateral; M, medial; P, posterior; V, ventral. Scale bar = 50 **(A–C)** and 25 μm **(D–F)**.

**FIGURE 4:**
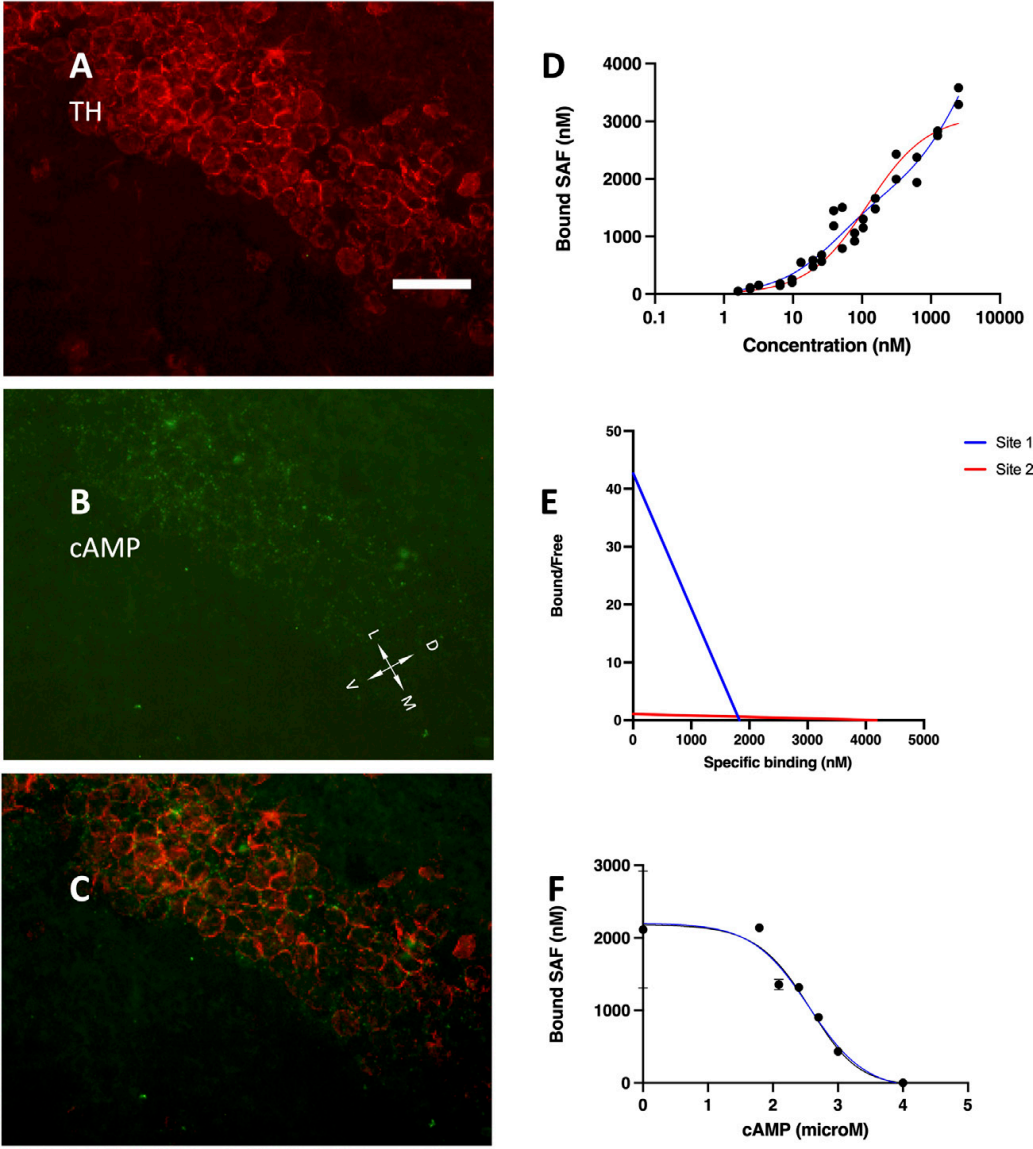
**(A)** tyrosine hydroxylase immunolabelling, mainly localized in the pyramidal layer. **(B)** Alexa488-cAMP labelling, same field as **(A)**. **(C)** merge of **(A,B)**; coronal section, 40× objective, D, dorsal; L, lateral; M, medial; V, ventral. Scale bar = 35 μm **(A–C)**. **(D)** SAF-cAMP equilibrium binding curve on CA1 pyramidal layer of a P30 rat. Red line: one-site nonlinear fit. Blue line: two-sites nonlinear fit. By increasing the concentration of SAF-cAMP (*x*-axis), the concentration in the fluorescent clusters increases up to 3,583 nM. **(E)** Scatchard plot of the same curve in **(D)**. **(F)** inhibition curve, SAF-cAMP 88 nM.

The development of hippocampus is protracted after birth, so that the different parts acquire their structure and function around puberty. Around 1 month of age in both rats and mice, CA1 pyramidal layer stands out with numerous small dot-like structures anchoring R1A, shown by immunofluorescence, which avidly binds fluorescent cAMP, shown by competitive binding (Figures 1A–F). Intense RIA and fluorescent cAMP labelling are localized within the pyramidal layer (Figures 1G–I). In high-power CA1 images, R1A and cAMP appear to colocalize (Figures 1J, K; see Figure 1L for localization of the area under investigation). Not all of these structures do dock the PKA catalytic subunit (Figures 2A–C, G–H), as already shown at a different magnification in Mucignat-Caretta and Caretta (2020). They appear to surround nuclei (Figures 2D–F), are prominent in CA1 pyramidal layer and absent in CA2, where PKA R2B is present, as detected by immunofluorescence, mostly in the stratum oriens (Figures 3A–C). Apparently, in CA1 cAMP clumps near cholinergic terminals (Figures 3D–F). No obvious colocalization is apparent with tyrosine hydroxylase (Figures 4A–C), a marker of catecholaminergic neurons.

### 3.2 Determination of cAMP binding properties by confocal microscopy

In order to visualize the fluorescently-tagged cAMP, its local concentration must exceed those of the background. The concentration of SAF-cAMP was estimated in confocal images under stringent conditions, including: non-saturating signals, no other possibly interfering fluorescent molecules, data collected where CA1 tissue is not bending (within area CA1B), data obtained in the range of linearity response curves of the photomultiplier tube; calibration performed before and after each experiment to account for possible laser power variations (see also Methods). Three independent experiments were run. By adding increasing amounts of SAF-cAMP, starting from 0.9 nM, the local concentration at each labelled structure increases up to more than 3,000 nM, as conservatively estimated by fluorescence measurements and assuming no or negligible short-range fluorescence interactions (Figure 4D). The curve is best fit by a two-sites interpolation (blue line in Figure 4D), which returns a maximum specific binding (BMax) for the two sites of 1,815 and 4,110 nM with equilibrium dissociation constants (Kd) of 23.55 and 2,094 nM, respectively. The one-site nonlinear fit instead returns Bmax = 3,104 nM with Kd = 67.79. A Hill slope of 0.595 suggests non-cooperativity of the two binding sites. The Scatchard plot confirms the lack of cooperativity between the binding sites (Figure 4E).

Competitive binding experiments were conducted using SAF-cAMP 88 nM, in order to have a sufficiently strong signal. The lowest concentrations were not used to avoid possible decrease in the concentration of free SAF-cAMP due to the avid binding to a very high number of binding sites, which are present not only on CA1 but also in other brain areas. The entire brain section was mounted in order to minimize cutting artifacts. By adding increasing quantities of unlabelled cAMP at the concentration indicated in Figure 4F (*X*-axis), the fluorescence measured from the labelled structures (Figure 4F, *Y*-axis), which peaks at more than 2000 nM, decreases and drop to zero, meaning that they become indistinguishable from the background, and return the equilibrium dissociation constant Ki = 22.9 nM.

## 4 Discussion

Hippocampus undergoes relevant changes in morphology and function during pre- and postnatal life with a protracted development, that in murine rodents extends up to the fourth postnatal week. It is noteworthy that this timing overlaps with the major changes in PKA regulatory subunits docking in the different subdivisions of hippocampus. There are multiple changes, both biochemical, electrophysiological and structural, occurring around this period of life, and some of them have been linked to PKA.

Hippocampal long-term memory and long-term potentiation functions rely on the cAMP/PKA second messenger. It acts both on the presynaptic terminal, by affecting different targets like RIM1alpha and synapsin, and on the postsynaptic cells, by acting mainly on glutamatergic AMPA and N-methyl-D-aspartate (NMDA) receptors to modulate membrane potential, and CREB to modulate gene expression (Abel and Nguyen, 2008). PKA is a relevant modulator of LTP in CA1 (Rodrigues et al., 2021). LTP involves Calcium influx through the NMDA glutamatergic receptor and subsequent PKA activation due to cAMP increase, in concert with phosphorylation of mitogen-associated protein kinase (MAPK) and cAMP-response element-binding protein (CREB). The neuronal cross-talk between cAMP/PKA to MAPK is critical for LTP plasticity to occur (Waltereit and Weller, 2003; Xia and Strom, 2012). Also, CB1 receptors induce the formation of inhibitory boutons via cAMP/PKA signaling (Liang et al., 2021), and novelty effects on memory rely upon D1 dopamine receptors that stimulate PKA (Lima et al., 2022).

PKA is thus critical for memory consolidation, at variance with its role in working memory (Arnsten et al., 2005). Dihydropyridine-sensitive calcium channels are affected by phosphorylation via PKA (Kavalali et al., 1997). Long-term depression in CA1 of two-week old mice is dependent on PKA to recruit AMPA receptors (Hell, 2016).

Hence, during the first weeks of life, different PKA-sensitive functions mature in hippocampus. It would be interesting to track the fate of cAMP dynamics within cells. The present data suggest that it is possible to explore the cAMP binding to PKA directly on tissue, to study the changes that could affect cAMP availability in subcellular microdomains and their developmental changes. This is intriguing given the different behavior of proteins in solution or docked in different conformation. Actually, the binding properties of the different PKA regulatory subunits in solution are quite similar (Mucignat-Caretta and Caretta, 1997), while the peculiar supramolecular organization of PKA R1, slowly emerging in CA1during postnatal development, shows an avid binding with biochemical properties which are different from those in solution, conceivably affecting the local concentration of cAMP and the temporal dynamics of its release.

Some hints can be drawn by the present data. The local concentration of cAMP at each single location is likely to modify cAMP gradients and local temporal dynamics of binding/release, thus buffering free cAMP and steeping gradients. Thus, cAMP availability may vary across development according to the different PKA regulatory subunit expressed at each site but also according to its supramolecular organization. As a suggestion, the appearance of R1A aggregates, possibly in cholinergic neurons (Mucignat-Caretta, 2000), can be related to the developmental maturation of memory functions: persistent firing within CA1 pyramidal neurons, a correlate of working memory, is sustained by cholinergic activation but suppressed by noradrenergic activation, which also acts through cAMP/PKA pathway (Valero-Aracama et al., 2021; Brahimi et al., 2023), so that developmentally regulated changes in cAMP buffering activity may help the emergence of mature memory processes. Cholinergic signaling, in cooperation with glutamatergic neurons, modulates also GABAergic inhibitory interneurons, whose activity is related to brain oscillatory patterns (Morales-Weil et al., 2020). Interestingly, the hippocampal GABAergic interneurons are rich in alpha7 nicotinic acetylcholine receptor, whose activation modulates both aggression (Lewis et al., 2018) and seizure susceptibility (Sun et al., 2021): PKA is among the interactome of this receptor (Paulo et al., 2009).

Despite the present data were obtained from brain sections, the calculated Ki for SAF-cAMP was in the nanomolar range, lower than those calculated for R1 in solution (Mucignat-Caretta and Caretta, 1997), 22.9 vs. 120 nM, respectively: the higher affinity may allow binding at lower cAMP free concentration, and subsequent PKA activation. A similar approach can be applied to living tissue slices and, using suitable fluorophores, could allow long-term monitoring of local circuits. Another issue concerns the study of fluorescence spectral and anisotropy changes due to binding of fluorescently-tagged cAMP. In solution, fluorescent cAMP 8-derivatives binding to PKA regulatory subunits not only affects fluorescence excitation/emission spectra, including red-shifts that were not studied here, but also affects fluorescence polarization (Mucignat-Caretta and Caretta, 1997), which is possibly related to nucleotide binding to slow or fast dissociating cAMP binding sites of PKA regulatory subunits. However, this different behavior of fluorescent cAMP is not an artifact due to modification at position 8, since 8-azido-[^32^P]cAMP both in solution and in the brain tissue do bind to both R1 and R2 (Mucignat-Caretta and Caretta, 2004). Hence, structural changes in the cAMP binding site or its accessibility do take place in specific brain sites at certain ages, and may be visualized with molecules whose steric hindrance is relatively large.

At the cell level, the functional consequences of changes in PKA binding ability are still unraveled, though the method to track the phenomena is a first step in this direction.

Structural changes support a different ability to bind cAMP at specific intracellular sites and developmental times: their impact on local cAMP dynamics and its downstream actions should be considered. This is further supported by the finding that fluorescently tagged cAMP, while binding only to R1 aggregates in the healthy brain, do bind to R2 in human meningioma samples (Caretta et al., 2019), raising the possibility that intracellular signaling may consistently be related to cell functional state and can be used for further deepen our understanding of cell functions in health and disease.

In conclusion, starting from the serendipitous observation that R1A clusters emerge slowly during development in CA1, in striking contrast with CA2, the present work suggests a method to track the cAMP binding activity to possibly relate it to functional properties emerging in the same timeframe.

## Data Availability

The original contributions presented in the study are included in the article/Supplementary Material, further inquiries can be directed to the corresponding author.
